# Induction of Tolerance via the Sublingual Route: Mechanisms and Applications

**DOI:** 10.1155/2012/623474

**Published:** 2011-11-10

**Authors:** Philippe Moingeon, Laurent Mascarell

**Affiliations:** Département Scientifique, Stallergènes SA, 6 rue Alexis de Tocqueville, 92160 Antony, France

## Abstract

The clinical efficacy of sublingual immunotherapy (SLIT) with natural allergen extracts has been established in IgE-dependent respiratory allergies to grass or tree pollens, as well as house dust mites. Sublingual vaccines have an excellent safety record, documented with approximately 2 billion doses administered, as of today, in humans. The oral immune system comprises various antigen-presenting cells, including Langerhans cells, as well as myeloid and plasmacytoid dendritic cells (DCs) with a distinct localisation in the mucosa, along the *lamina propria* and in subepithelial tissues, respectively. In the absence of danger signals, all these DC subsets are tolerogenic in that they support the differentiation of Th1- and IL10-producing regulatory CD4^+^ T cells. Oral tissues contain limited numbers of mast cells and eosinophils, mostly located in submucosal areas, thereby explaining the good safety profile of SLIT. Resident oral Th1, Th2, and Th17 CD4^+^ T cells are located along the *lamina propria*, likely representing a defence mechanism against infectious pathogens. Second-generation sublingual vaccines are being developed, based upon recombinant allergens expressed in a native conformation, possibly formulated with Th1/T reg adjuvants and/or mucoadhesive particulate vector systems specifically designed to target oral dendritic cells.

## 1. Introduction

Following the pioneer studies by Noon and Freeman [1, 2] conducted a century ago, allergen-specific immunotherapy is presently the only curative treatment for type I allergies. Specifically, subcutaneous immunotherapy (SCIT) was shown to provide clinical benefit for patients with IgE-dependent allergies to either grass, weed, and tree pollens, dust mites (e.g., *Dermatophagoides pteronyssinus, Dermatophagoides farinae*), cat and dog epithelia, or moulds [3, 4]. Also, SCIT has become a reference treatment for venom allergies [3]. Although SCIT has been occasionally performed with soluble allergens, in most circumstances, subcutaneous vaccines include adjuvants such as aluminum hydroxide or calcium phosphate. Since SCIT requires multiple injections and can be associated with severe side effects, including anaphylactic shocks, safer and noninvasive mucosal routes of administration have been explored as an alternative [5, 6]. 

Most particularly, sublingual immunotherapy (SLIT) was investigated in allergic patients almost twenty years ago and is now established as a valid noninvasive alternative to subcutaneous immunization to treat type I respiratory allergies [5–8]. Although the sublingual route is the only mucosal route commonly used in humans for tolerance induction in allergic patients, other exploratory routes are being tested, including the oral, intranasal, epicutaneous and intralymphatic routes [9–20] (Table 1). This review focuses on the clinical indications, mechanisms of action, and future developments pertaining to sublingual allergy vaccines.

## 2. Sublingual Allergy Vaccines as a New Therapeutic Class

Sublingual immunotherapy (SLIT) represents a form of therapeutic vaccination aiming to a long-term allergen-specific immunomodulation in patients with allergic rhinoconjuctivitis, with or without moderate asthma [6]. It is performed by reiterated administration (over months or even years) of an allergen extract in the form of drops, fast dissolving tablets, or lyocs [6, 21]. Patients are asked to maintain the allergen(s) under the tongue for 1-2 mn to allow contact with the oral mucosa. The allergens are subsequently swallowed, as per the “sublingual-swallow” procedure. In all circumstances, high doses (usually 50- to 100-fold the ones used for SCIT) of the allergen(s) are being used in the absence of any adjuvant [6, 21]. Currently, sublingual vaccines are based on aqueous extracts prepared from natural allergenic materials such as animal hair, pollens, or lab cultures of house dust mites [22]. Less frequently, these allergens are treated with glutaraldehyde to form polymers precluding IgE reactivity, but the clinical efficacy of such “allergoïds” remains to be documented [3, 4]. 

Multiple studies further compiled in metaanalyses have demonstrated the efficacy of sublingual drops in adult and pediatric patients with allergic rhinoconjunctivitis to either pollens (from common grasses, ragweed, parietaria, birch, olive tree, cupressus), house dust mites, or cat dander [23–27]. More specifically, SLIT significantly reduces both rhinoconjunctivitis symptoms as well as the need for symptomatic medication. These studies, as well as the experience cumulated by allergists during their daily practice (which together account for around 2 billion doses of sublingual vaccines administered to human beings), have unambiguously documented the excellent safety profile of sublingual vaccines [6, 23–27]. Specifically, treatment-related adverse events include frequent (i.e., in more that 60% of patients) but moderate local reactions in the form of throat irritation, ear pruritus, or tongue oedema [6]. Those adverse events are mostly observed when SLIT is initiated, and are usually self resolving without any specific further treatment. In contrast to SCIT, systemic reactions, most particularly in the form of anaphylaxis, are extremely rare, and are thought to be linked to the nonrespect of commonly accepted medical guidelines (i.e., administration of overdoses or inappropriate allergen mixes, treatment of patients with uncontrolled asthma) [4, 6]. 

Recently, two sublingual grass pollen tablets have been developed and shown in multiple double-blind placebo-controlled Phase III clinical studies conducted in both adults and children to be highly efficacious, with an overall 30–40% improvement in rhinoconjonctivitis symptom scores when compared with placebo [28, 29]. Another large-scale study has also documented the clinical efficacy of SLIT in perennial allergy (i.e., to house dust mites) using a tablet containing extracts from the two common *D. pteronyssinus *and* D. farinae* mite species [30]. Other trials have demonstrated a long-term efficacy of sublingual immunotherapy, for example, following a three-year administration of grass pollen tablets [31, 32]. In addition, those allergic patients remained protected for at least two years after stopping the treatment, thus documenting a “disease-modifying” effect of SLIT. Based on those results, such sublingual tablets have been registered as pharmaceutical specialties (Grazax, Oralair) in Europe. The recommendation to use sublingual immunotherapy has been endorsed by the World Health Organisation (WHO) in several position papers on allergen immunotherapy as well as by the allergic rhinitis and its Impact on asthma (ARIA) workshop group [3, 4, 6].

## 3. Specific Properties of the Oral Immune System

**Figure 1:**
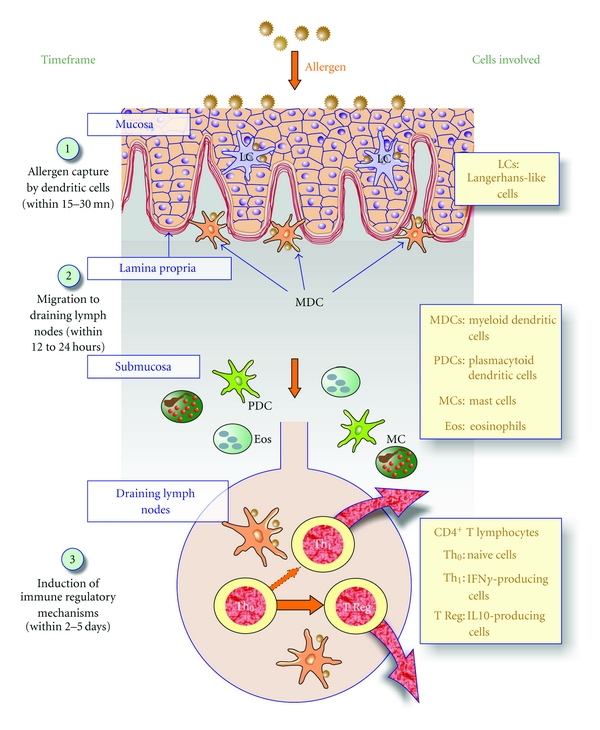
Fate of the allergen following sublingual administration. Following sublingual immunization, substantial amounts of the allergen bind to epithelial cells within minutes, then cross the mucosa between 15 and 30 minutes. The allergen is subsequently captured by dendritic cells (likely by Langerhans cells (LCs) within the mucosa itself and myeloid dendritic cells (MDCs) along the lamina propria) and processed as small peptides presented in association with MHC class I and class II molecules at the cell surface. Those DCs loaded with allergen-derived peptides reach cervical lymph nodes within 12 to 24 hours, where they interact with naive CD4^+^ T cells to support the differenciation of Th1 and T Reg cells within two to five days. These CD4^+^ T cells subsequently migrate into the blood and back to mucosal tissues, resulting in allergen tolerance with downregulation of preexisting Th2 responses.

The sublingual route has been initially used for small synthetic drugs (e.g., nitroglycerine, opioid analgesics) for which a fast plasmatic release was needed [21]. In contrast to such small molecules, proteins do not cross the mucosa to reach the bloodstream, but are rather captured by professional antigen-presenting cells (APCs) within 15 to 30 minutes, which will subsequently migrate to draining cervical submaxillary lymph nodes within 12 to 24 hours (Figure 1) [21, 33]. This makes the sublingual route very interesting for clinical tolerance induction over other mucosal routes, including the oral route, in that the antigen is being captured and processed by APCs prior to significant proteolytic degradation, thus preserving the integrity of T and B cell epitope repertoires.

A detailed mapping of the oral immune system, most particularly of antigen-presenting cells (APCs), has been completed in mice [33, 34]. Specifically, three subsets of oral dendritic cells (DCs) exhibiting a distinct tissue distribution have been identified including (i) Langerhans cells (LCs) located in the mucosa itself, (ii) a predominant subpopulation of myeloid DCs (MDCs) located along the *lamina propia,* and (iii) plasmacytoid DCs (pDCs) found in submucosal tissues (Figure 1) [33]. In humans, LCs have similarly been described in the mucosa itself, whereas myeloid and plasmacytoid DCs are less abundant [35–38]. Noteworthy, all these DC subsets are thought to be tolerogenic, in that they produce both IL-10 and IL-12 cytokines and thus, drive the differentiation of naïve CD4^+^ T cells towards a Th1/T Reg phenotype (Figure 1). Among those APCs, Langerhans cells and a subset of macrophage-like CD11b+CD11c− APCs are thought to be critical in capturing the antigen/allergen [33, 35, 36, 39].

Only few proinflammatory cells (i.e., mast cells (MCs) or eosinophils (Eos)) are found in oral tissues, and these cells are mostly located in muscular tissues (Figure 1) [34, 38]. In this context, most allergens are likely captured by tolerogenic dendritic cells in the upper layers of oral tissues prior to reaching proinflammatory mast cells, thus explaining the excellent safety profile of the sublingual route, with virtually no risk of severe systemic reactions when compared with the subcutaneous route [6, 21, 35]. Lastly, resident CD4^+^ T lymphocytes are abundant in oral tissues, located in the vicinity of myeloid APCs along the *lamina propria*. These cells comprise both suppressive as well as effector T cells, including Th1, Th2, and Th17 lymphocytes, likely involved in defence against infectious pathogens. This explains why the sublingual route is also currently considered to elicit effector immune responses against pathogenic viruses [40]. Nonetheless, in the absence of any danger signal, the default response to an antigen administered via the sublingual route is tolerance induction following the induction of Th1/T reg CD4^+^ T cells [21, 35].

## 4. Immune Changes Associated with Sublingual Immunotherapy 

Allergen-specific immunotherapy is known to reduce both immediate- and late-phase allergen-induced symptoms, via both humoral and cellular mechanisms [41–43]. Immune mechanisms leading to clinical tolerance, described in more details below, are thought to be associated with both subcutaneous and sublingual immunotherapy. Most particularly, changes in the polarization of allergen-specific CD4^+^ T-cell responses are considered to be central, in that variations in the patterns of cytokines produced significantly impact antibody responses as well as recruitment and activation of proinflammatory cells in target mucosae [21, 41–43]. 

### 4.1. Antibody Responses

After an initial rise, allergen-specific sublingual immunotherapy induces a prolonged decrease in seric IgE levels and prevents the seasonal increase in IgEs associated with exposure to environmental allergens [21, 41–43]. For example, pollen immunotherapy results in the blunting of the seasonal upregulation of specific IgEs, while eliciting allergen-specific IgG responses—mostly IgG1 and IgG4. Such IgG antibodies may act as “blocking” antibodies by competing with IgEs for allergen binding, thereby inhibiting IgE-mediated allergen presentation to T cells [44]. In addition, they can engage low-affinity Fc receptors for IgGs (CD32) known to down regulate mast cell and B cell activation [45]. A specific property of SLIT, when compared to SCIT, is further to elicit allergen-specific IgAs, both in serum and mucosal secretions [43, 46, 47].

### 4.2. Proinflammatory Cells

A reduced recruitment and activation of inflammatory cells in target mucosae has been observed following allergen-specific immunotherapy [48–50]. Specifically, successful SLIT has been associated with a decrease in the recruitment of mast cells (both tryptase/chymase+ or tryptase+ only), basophils, and eosinophils in the skin, nose, eye, and bronchial mucosae [21, 43].

### 4.3. T-Cell Responses

Sublingual immunotherapy shifts allergic-specific CD4^+^ T-cells responses from Th2 to Th1, with the stimulation of IFN*γ*-producing T lymphocytes [21, 42, 43]. In addition, SLIT also induces regulatory T (T Reg) cells, thought to play a central role in inhibiting effector mechanisms associated with allergic inflammation [51, 52]. T Reg cells induced during immunotherapy are type 1 (Tr1) cells producing high levels of IL-10 and/or transforming growth factor-*β* (TGF-*β*), known to decrease IgE production and to enhance IgG4 and IgA production, respectively [42, 43]. In addition, both IL-10 and TGF-*β* lower the release of proinflammatory mediators and inhibit the production of Th2 cytokines [42].

## 5. Future Directions

### 5.1. Development of Sublingual Recombinant Allergy Vaccines

There is, as of today, no recombinant allergy vaccine commercially available. With the advent of molecular biology and the use of recombinant DNA technology, the possibility of developing highly purified allergens for sublingual immunotherapy is raising considerable interest [22, 53]. Over the last decade, genes encoding the most important allergens have been cloned (cf., updated list at http://www.allergen.org/) and for a number of them, expressed as recombinant proteins. Such recombinant allergens offer the advantage over natural extracts of being better characterized and easier to produce in a consistent manner [22, 53].

Vaccines based on recombinant allergens are particularly suitable when the number of target allergens is limited, which is the case for birch (*Betula verrucosa*) pollen allergy [54]. Over 95% of patients allergic to birch pollen display IgE reactivity to the Bet v 1 allergen and up to 60% of these patients are sensitized to Bet v 1 only. A recombinant form of Bet v 1 (isoform a) has been produced in *Escherichia coli *and shown to be folded similarly to the native protein, with a compact and stable structure and a well-preserved antigenicity [54]. This rBet v 1 protein has been initially tested in a Phase I/II clinical trial via the subcutaneous route (using 15 ug/dose in association with Alum as an adjuvant). Under those conditions, the rBetv 1 allergen alone was as efficient as the total birch pollen extract in alleviating patients' symptoms during the pollen season [55]. Based on those results, rBet v 1 has been administered without any adjuvant via the sublingual route in a Phase II study and shown to decrease significantly rhinoconjunctivitis symptoms as well as the use of symptomatic medications, in comparison to placebo [56]. 

One pending question regarding the use of recombinant allergens for immunotherapy is whether IgE binding epitopes should be preserved in the molecule [53]. Hypoallergenic forms of recombinant allergens or peptide fragments have been produced which do not induce degranulation of IgE+ mast cells or basophils, while maintaining their capacity to elicit IgG and CD4^+^ T responses [53]. However, while hypoallergens could in theory represent safer vaccines, there is, as of today, no evidence of their clinical efficacy. With respect to the sublingual route, oral DCs have been shown to express Fc receptors for IgEs, which thus can be used to better address the allergen onto APCs [37]. Interestingly, in the Phase II study described above, rBet v 1 administered sublingually was well tolerated at doses up to 50 ugs, besides the expected local reactions commonly associated with SLIT [56]. For those reasons combined, our working hypothesis is that recombinant allergens to be used sublingually should rather be produced in a wild-type (i.e., native) conformation in order to mimick the natural allergen [21, 22]. 

### 5.2. Adjuvants and Vector Systems for Sublingual Vaccines

Novel adjuvants and vector systems could be considered to further improve the efficacy of sublingual allergy vaccines [57]. Those immunopotentiators could as well allow to reduce the dose of allergens or simplify immunization schemes. Potential mucosal adjuvants which have been successfully tested in murine SLIT models to modulate T cell polarization include ligands for Toll-like receptors (TLRs) 2 (e.g., Pam3Cysk4) and 4 (i.e., synthetic lipid A analogs) [57–59]. Such TLR ligands enhancing tolerance induction via the sublingual route share in common a capacity to elicit mixed Th1/T reg CD4^+^ T cell responses. In addition, dexamethasone + (1, 25) dihydroxyvitamin D3 as well as selected strains of probiotics (i.e., lactobacilli, bifidobacteria) represents potential T reg adjuvants, since they are powerful inducers of IL-10 production by immune cells. As such, these compounds enhance SLIT efficacy in murine models of OVA-induced asthma [58–61]. In humans, the only adjuvant which has been tested via the sublingual route is monophosphoryl lipid A (MPL), a TLR4 ligand-inducing Th1 responses. Coadministration of MPL enhanced IgG responses to the allergen when using high doses of adjuvant [62]. The clinical relevance of this enhancement of specific antibody responses remains to be established.

Mucosal vectors could also enhance SLIT efficacy, for example, by protecting the allergen(s) from degradation by local proteases or by targeting the allergen to antigen-presenting cells [57]. As an example, the genetically detoxified adenylate cyclase (CyaA) from *Bordetella pertussis* conjugated to OVA was shown to enhance tolerance induction via the sublingual route in OVA-induced asthmatic mice, as a consequence of a superior targeting of oral CD11b+ tolerogenic myeloid DCs [39]. In addition, positively charged polymers have been used to generate mucoadhesive particulate vectors which can enhance allergen interaction with negatively charged epithelial cells and as a consequence, contact duration with the mucosa. Formulations of allergens within a particle increase allergen uptake by antigen-presenting cells with a phagocytic activity [57]. For example, both nanoparticles made from polymerized maltodextrin [47] or chitosan-based microparticles [63] were found to enhance *in vitro *and *in vivo* allergen capture by tolerogenic oral DCs, thus resulting in a stronger tolerance induction via the sublingual route in murine asthma models. To date, no vector system has been evaluated sublingually in humans.

### 5.3. New Clinical Indications

Both subcutaneous and sublingual immunotherapies of patients with rhinoconjunctivitis appear to prevent subsequent sensitization and evolution towards asthma [24, 25, 27, 64]. In addition, several studies suggest a benefit of SLIT in controlling asthma associated with house dust mites [6, 65, 66]. Additional clinical trials in adult and pediatric patient populations are needed to further document a benefit of SLIT in this indication. Recently, SLIT has also been tested successfully in several new clinical indications, including allergies to latex and food (e.g., peach, kiwi, hazelnut) [67–69]. These studies conducted on small cohorts of patients provided encouraging results, both in terms of safety and clinical efficacy (e.g., increase in amounts of food allergens tolerated by the patients). Similarly, SLIT has been recently shown to decrease atopic dermatitis symptoms linked with mite exposure, in patients with mild-to-moderate disease [70]. Such results need to be further confirmed in the context of large-scale-double blind placebo-controlled studies.

## 6. Conclusions

Sublingual vaccines based on biological extracts are being used as a safe and efficacious treatment for type I respiratory allergies. To provide consistent pharmaceutical-grade products despite the inherent variability associated with biological extracts, well-established standardisation procedures and comprehensive proteomic characterization methods are being used to guarantee the quality of allergen extracts and the robustness of manufacturing processes. Those improvements have been recognised by regulatory authorities with the registration in 2008 of sublingual grass pollen tablets as pharmaceutical specialties. New applications are being pursued, encompassing the development of sublingual tablets for mite and ragweed pollen allergies, as well as the evaluation of SLIT as a treatment of asthma. Additional frontiers to explore in the long term include the development of sublingual vaccines for food allergies and atopic dermatitis. In parallel, second-generation vaccines based on recombinant allergens are being investigated to treat birch pollen allergies. These vaccines will associate recombinant allergens in a native conformation, together with Th1/T Reg adjuvants and/or mucoadhesive particulate vector systems. If successful, such recombinant sublingual vaccines could enhance clinical efficacy while reducing treatment duration and decreasing the dose of allergen administered.

## Figures and Tables

**Table 1:** Compared characteristics of sublingual versus other administration routes for allergy vaccines.

Routes	Current clinical indications	Comments	Ref
Sublingual (SLIT)	(i) Established as a safe and efficacious treatment for IgE-dependent respiratory allergies (rhinoconjunctivitis with or without moderate asthma) (ii) For adults and 5–15 year old children	(i) No adjuvants (ii) Dose 50 to 100 fold the one used for SCIT (iii) Treatments available as drops, fast-dissolving tablets, lyocs) (iv) Two sublingual grass pollen tablets (Grazax, Oralair) have been registered in Europe as pharmaceutical specialties) (v) Excellent safety record (mostly moderate local reactions). Systemic reactions are extremely rare) (vi) Efficacy documented by large scale double blind placebo controlled Phase III trials (evidence-based medicine)	[5–8]

Subcutaneous	(i) Same as SLIT (ii) Venom allergies (iii) Latex allergies	(i) Adjuvants (aluminum salts or calcium phosphate) are being used (ii) For effective immunotherapy, a 5 to 25 *μ*g maintenance dose of allergen is necessary (iii) Efficacy documented by historical practice (reference route since 1911) (iv) Potential safety issues (besides acceptable local reactions, risk of infrequent but life threatening anaphylactic shocks).	[3, 4]

Exploratory routes (oral, nasal, epicutaneous, intralymphatic)	(i) None as of today (ii) Numerous clinical studies are being conducted in patients with respiratory allergies (mites, pollens) or food allergies (milk, egg, peanut)	(i) Encouraging results in small cohorts of patients (ii) Safety and efficacy remain to be confirmed in large scale clinical studies. (iii) Expected positive outcomes of those new routes include new applications for immunotherapy (e.g., food allergy for the oral or epicutaneous routes) or tolerance induction with a limited number of administrations (e.g., intralymphatic route)	[9–20]
